# Data on tensile tests and NMR measurements of poly(vinilidene fluoride) before and after stress relaxation

**DOI:** 10.1016/j.dib.2018.04.145

**Published:** 2018-05-04

**Authors:** Maria Marjorie Contreras, Christine Rabello Nascimento, Roberto Pinto Cucinelli Neto, Marysilvia F. Costa, Celio A. Costa

**Affiliations:** a Programa de Engenharia Metalúrgica e de Materiais, Universidade Federal do Rio de Janeiro, Rio de Janeiro 68505, Brazil; b Instituto de Macromoléculas Professora Eloisa Mano, Universidade Federal do Rio de Janeiro, Rio de Janeiro 68525, Brazil

## Abstract

Poly(vinilidene fluoride) was characterized before and after stress relaxation by tensile tests and time-domain nuclear magnetic resonance (TD-NMR). Tensile tests were performed to provide mechanical properties, focused on the data of elastic modulus for this matter. The TD-NMR technique was used to calculate the fraction of crystalline, constrained amorphous and free amorphous phase, and the transversal relaxation time of each of these phases.

**Specifications Table**

**Table:** 

Subject area	Material science engineering.
More specific subject area	Polymers, Characterization, Mechanical behavior.
Type of data	Table and data files
How data was acquired	Tensile tests: universal testing machine (Instron 5567), TD-NMR: MARAN Ultra spectrometer with an electromagnetic field of 0.54 T (23.4 MHz for ^1^H) and probe diameter of 18 mm.
Data format	Raw and analyzed data.
Experimental factors	Samples were relaxed at three different temperatures (23, 80 and 120 °C) and, for each temperature, three different strains (3.5, 7 and 10%) during 24 h and loaded with a crosshead speed of 5 mm.min^−1^.
Experimental features	Tensile tests were carried out with crosshead speed of 5 mm.min^−1^ at 23 °C. TD-NMR measurements were conducted at 28 °C.
Data source location	Laboratorio de Processamento e Caracterização de Materiais (LPCM/COPPE/UFRJ); Universidade Federal do Rio de Janeiro, Rio de Janeiro, Brazil.
Data accessibility	The dataset is available in this article.
Related research article	[1]

**Value of the data**

• The following data allowed to correlate the elastic modulus and structural evolution of. poly(vinilidene fluoride) before and after stress relaxation, a routine condition imposed in flexible pipe fabrication, storage and operation.
• The data was collected on up to date equipments, with very good resolution, allowing a more consistent analysis.
• It permitted to interpret the effect of chain displacement on the intrinsic stiffness, represented by the elastic modulus, due to the stress relaxation process.
• The data can be helpful for identifying structural evolution on materials and the importance of reevaluating test procedures because nanoscale damage in polymeric materials may start at small strain, short time and low temperature.

## 1 Data

This data article contains tables and data files (raw data) showing elastic modulus and TD-NMR measurements of poly(vinilidene fluoride) (PVDF) before and after stress relaxation.

Table 1 presents values of average of elastic modulus (3 samples for each condition) for as processed material and all stress relaxed conditions. The TD-NMR results are shown in Table 2 (one sample per condition), and it reported the fraction and transversal relaxation time of each region of the material structure, namely: crystalline, constrained amorphous and free amorphous phases. To determine these parameters, values of time and amplitude (in Supplementary Table S1) obtained by TD-NMR were analyzed.

**Table 1:** Elastic modulus from tensile tests.

Sample	Elastic modulus (MPa)
PVDF	1292.7 (± 97.9)
PVDF relaxed at 23 °C, 3.5% ε_0_	951.4 (± 40.1)
PVDF relaxed at 23 °C, 7% ε_0_	797.3 (± 3.4)
PVDF relaxed at 23 °C, 10% ε_0_	837.1 (± 70.7)
PVDF relaxed at 80 °C, 3.5% ε_0_	942.7 (±73.8)
PVDF relaxed at 80 °C, 7% ε_0_	868.2 (± 49.2)
PVDF relaxed at 80 °C, 10% ε_0_	899.7 (± 68.2)
PVDF relaxed at 120 °C, 3.5% ε_0_	724.4 (± 16.7)
PVDF relaxed at 120 °C, 7% ε_0_	730.4 (± 16.9)
PVDF relaxed at 120 °C, 10% ε_0_	761.0 (± 39.6)

**Table 2:** TD-NMR measurements obtained by MSE-FID technique.

Sample	F_CRYS_^a^ (%)	T_2_* _CRYS_^b^ (μs)	F_CONST_^a^ (%)	T_2_* _CONST_^b^ (μs)	F_AM_^a^ (%)	T_2_* _AM_^b^ (μs)
PVDF	38	21	41	149	23	512
PVDF relaxed at 23 °C, 3.5% ε_0_	26	20	51	104	22	395
PVDF relaxed at 23 °C, 7% ε_0_	23	19	55	101	22	385
PVDF relaxed at 23 °C, 10% ε_0_	35	20	44	138	23	521
PVDF relaxed at 80 °C, 3.5% ε_0_	24	21	52	98	24	405
PVDF relaxed at 80 °C, 7% ε_0_	26	20	54	112	21	484
PVDF relaxed at 80 °C, 10% ε_0_	34	21	48	141	20	553
PVDF relaxed at 120 °C, 3.5% ε_0_	20	22	59	86	20	405
PVDF relaxed at 120 °C, 7% ε_0_	28	21	52	103	19	428
PVDF relaxed at 120 °C, 10% ε_0_	26	21	54	101	19	396

ε_0_: strain of the stress relaxation test.

a Standard deviation ± 2 °C.

b Standard deviation ± 5 μs.

## 2 Experimental design, materials, and methods

Flexible lines may have PVDF material in the internal pressure sheath and it is submitted to stress relaxation during fabrication, storage and operation (oil and gas offshore industry). Nonetheless, the properties-structural evolution relationship after stress relaxations was not found in the literature by the authors. PVDF was characterized by tensile tests and TD-NMR measurements before and after stress relaxation process.

### 2.1 Materials

PVDF samples were prepared to a previously reported method [1].

### 2.2 Tensile tests

Tensile tests were carried out on a universal test machine (Instron 5567) according ASTM D 638 [2]. The specimens were placed in the grips of the test machine, at a specified grip separation and pulled until failure. The tests were conducted with a crosshead speed of 5 mm min^−1^ at 23 °C. Stress-strain curves were plotted and the elastic modulus was calculated from the slope of the initial part of the stress-strain curve in strain range between 0.05% and 0.5%.

### 2.3 TD-NMR experiments

The TD-NMR measurements were performed on a MARAN Ultra spectrometer with an electromagnetic field of 0.54 T (23.4 MHz for ^1^H) at 28 ± 2 °C and probe diameter of 18 mm. The samples were analyzed using the free induction decay refocused through magic sandwich echo technique (MSE-FID) [1], [3], [4], [5], [6]. This sequence consisted of a pulse 90° followed by a period of evolution τ_MSE_ (33 μs), and the central part was formed by a symmetrical standard of 8 pulses 90°. Then, other pulse 90° was applied and the same τ_MSE_ from the beginning of the sequence before the acquisition of the signal was waited. Values of amplitude versus time obtained from these measurements were presented in the Supplementary Table S1. The normalized MSE-FID signals were fitted by Eq. (1) [1].The signal obtained is composed of three different regions: the crystalline region that obeys an Abragamian function, the constrained amorphous phase (or intermediate region) fitted by a Gaussian function, and the free amorphous region (or higher mobility phase) which exhibited a decay of exponential behavior.

(1) $$A\left(t\right)=F_{\mathrm{CRYS}}.\exp\left[-\frac{1}{2}{\left(\frac{t}{T_{2*-\mathrm{CRYS}}}\right)}^{2}\right].\left(\frac{\sin(2\pi \nu t)}{2\pi \nu t}\right)+F_{\mathrm{CONST}}.\exp\left[-{\left(\frac{t}{T_{2*-\mathrm{CONST}}}\right)}^{\beta_{i}}\right]\;\;\;\;\;\;{+F}_{\mathrm{AM}}.\exp\left[-{\left(\frac{t}{T_{2*-\mathrm{AM}}}\right)}^{\beta_{a}}\right]+k$$

where A is amplitude, t is time. F_CRYS_, F_CONST_ and F_AM_ (%) are amplitudes or fractions of crystalline, constrained amorphous and free amorphous regions, respectively. T_2_* is the transversal relaxation time of each of these regions, ν is the sinusoidal oscillation constant of the rigid (crystalline) component based on the second and fourth moments of Van Vleck, βi and βa are shape parameters, and k is the offset or baseline of the relaxation signal that compensates for the influence of noise during non-linear adjustment.

The fraction (%) of each region is calculated using the Eq. (2) [1].

(2) $$\mathrm{Fraction}=\frac{F}{F_{\mathrm{CRYS}}+F_{\mathrm{CONST}}{+F}_{\mathrm{AM}}}.100$$

$$F=F_{CRYS},F_{CONST}\;or\;F_{AM}.$$
