# Supplementary material for: Data on tensile tests and NMR measurements of poly(vinilidene fluoride) before and after stress relaxation
Source: Data Brief. 2018 May 4;19:55–8. doi: 10.1016/j.dib.2018.04.145 (PMC5992969; doi:10.1016/j.dib.2018.04.145)
Supplement: Supplementary file 1 — Supplementary material [file mmc1.pdf]

COPPE

LPCM
Laboratório de Processamento
e Caracterização de Materiais
COPPE | Poli | UFRJ

Rio de Janeiro, April 18, 2018

Managing Editor –
Co-EiC
Data in Brief

Ref: DIB-D-18-0026 - Conflict of Interest

Title: Data on tensile tests and NMR measurements of poly(vinilidene fluoride) before and after stress relaxation

Dear Editor,

I, hereby, representing all authors listed in this publication guarantee that the data and the respective knowledge herein, have no Conflict of Interest, whatsoever, we companies, governments and/or financial supporting agencies. It is an academic study.

Sincerely,

Celio A Costa, Ph.D.
